# To Explore the Key Active Compounds and Therapeutic Mechanism of Guizhi Gancao Decoction in Coronary Heart Disease by Network Pharmacology and Molecular Docking

**DOI:** 10.1155/2022/2566407

**Published:** 2022-11-23

**Authors:** Hua-jing Yuan, Yi-tao Xue

**Affiliations:** ^1^Shandong University of Traditional Chinese Medicine, Jinan 250014, China; ^2^Affiliated Hospital of Shandong University of Traditional Chinese Medicine, Jinan 250014, China

## Abstract

**Objective:**

Coronary heart disease (CHD) is the leading cause of death from cardiovascular disease and has become an important public health problem worldwide. Guizhi Gancao Decoction (GGD) has been shown to be used in the treatment of CHD with good efficacy, but its specific therapeutic mechanism and active ingredients have not been fully clarified. This study aims to identify the active compounds and key targets of GGD in the treatment of CHD, explore the therapeutic mechanism of GGD, and provide candidate compounds for anti-CHD drug development.

**Methods:**

The main compounds of GGD were determined by UPLC-MS/MS analysis and screened by SwissADME. The corresponding targets of GGD compounds were obtained from SwissTargetPrediction, and the targets of CHD were obtained from the HERB and GeneCards databases. The STRING 11.5 database was used to analyze the PPI (Protein-Protein Interactions) network of potential therapeutic targets of GGD compounds. Cytoscape 3.7.2 was used to construct target-related networks and find core targets. The GEO database was used to validate the differential expression of core targets. The PANTHER Classification System was used to functionally classify potential therapeutic targets for GGD. The GO biological process analysis and KEGG pathway analysis of targets were completed by DAVID 6.8 database. AutoDockTools 1.5.6 and PyMol 2.5.2 were used to perform molecular docking of core targets with the active GGD compounds.

**Results:**

7 active GGD compounds were obtained based on UPLC-MS/MS and pharmacological parameter evaluation, which corresponded to 131 CHD-related targets. Among them, EGFR, MAPK3, RELA, CCND1, ESR1, PTGS2, NR3C1, CYP3A4, MMP9, and PTPN11 were considered core targets. According to the targets related to CHD, glycyrrhetinic acid, liquiritigenin, and schisandrin are considered key active ingredients. GO biological process and KEGG analysis indicated that the potential targets of GGD in the treatment of CHD involve a variety of biological processes and therapeutic mechanisms. Molecular docking results showed that both the core targets and the corresponding compounds had the good binding ability.

**Conclusions:**

This study contributes to a more comprehensive understanding of the therapeutic mechanism and active ingredients of GGD for CHD and provides candidate compounds for drug development of CHD.

## 1. Introduction

Coronary heart disease (CHD) is a group of clinical syndromes caused by the development of coronary atherosclerosis. It can be clinically manifested as chest tightness, chest pain, and insufficient exercise endurance. CHD is a common cause of death from cardiovascular disease (CVD), accounting for 20% of deaths in Europe according to an epidemiological report in 2016 [1]. Therefore, CHD has become an important public health problem worldwide, which not only affects people's quality of life but also increases social and economic burdens [2]. At present, the therapeutic mechanism for CHD is not only to reduce the degree of coronary stenosis and delay the process of atherosclerosis, but also to improve and reverse ventricular remodeling, reduce myocardial ischemia-reperfusion injury (MIRI), and avoid myocardial necrosis. And the long-term use of antiplatelet and statin drugs may have adverse effects on the human body [3–5]. Therefore, there is a need to find new, low-risk alternative treatments for CHD.

Traditional Chinese medicine (TCM) has a history of thousands of years. It uses natural medicinal plants and has the advantages of good curative effects and low side effects. Guizhi Gancao Decoction (GGD), composed of *Ramulus cinnamomi* (Guizhi) and *Radix glycyrrhizae* (Gancao), has long been used to treat a variety of CVDs including CHD, arrhythmias, and heart failure. CHD belongs to the category of “chest stuffiness and pains” in TCM, which manifests as chest tightness, chest pain, shortness of breath, and fatigue. TCM believes that the lack of yang qi in the heart is its basic pathogenesis, and clinical treatment focuses on warming yang and replenishing qi, promoting blood circulation and dredging the pulse. GGD has the effect of warming and nourishing the yang qi of the heart, dredging collaterals. Current studies have shown that GGD can inhibit inflammation and myocardial apoptosis, prevent myocardial systolic and diastolic dysfunction, reduce MIRI, and has a significant cardiac protection effect [6, 7]. However, due to the characteristics of multi-component and multi-target action of TCM compounds, their biological effects are not clear.

Network pharmacology can display the “drug-compound-target” network to better reflect the relationship and the mechanism of action between drugs and diseases [8]. Therefore, this study explored the effective compounds and therapeutic mechanism of GGD in CHD through network pharmacology. We obtained the active compounds of GGD from the study of ultra-performance liquid chromatography-tandem mass spectrometry (UPLC-MS/MS) [9], which improved the accuracy of the study. In addition, we also used molecular docking to validate the targets of GGD for the treatment of CHD.

## 2. Materials and Methods

### 2.1. Determination of Main Active Compounds of GGD and Evaluation of Pharmacological Parameters

A total of eight active compounds in plasma were obtained from the UPLC-MS/MS study of GGD [9]. Lipinski's Rule of Five (RO5) is an important prerequisite for screening drug-like compounds. Compounds that comply with RO5 are considered to have better pharmacokinetic properties and bioavailability and are also more likely to be oral drugs [10]. The web tool SwissADME (https://www.swissadme.ch) [11] was used to evaluate the pharmacokinetic properties and compliance with the RO5 principle of GGD active compounds. Drug toxicology is an integral part of clinical research and new drug development. The ProTox-II webserver (https://tox-new.charite.de/protox_II/) [12] was used to predict the toxicity of compounds.

### 2.2. Data Collection of the Targets

The targets of GGD compounds were obtained by SwissTargetPrediction (https://www.swisstargetprediction.ch/) [13]. Specifically, we retrieved Canonical SMILES of GGD compounds in the PubChem database (https://pubchem.ncbi.nlm.nih.gov/) and entered them into SwissTargetPrediction, the target species was set to Homo sapiens, and according to the median of probability, values to obtain compound targets.

Bencao Zujian (HERB, https://herb.ac.cn/) [14] is a high-throughput database that manually extracted 494 diseases and 1241 gene targets from 1966 unique articles. And GeneCards (https://www.genecards.org/) [15] is a comprehensive and integrative database that provides annotated and predicted human genetic information. So we searched the database with the keyword “coronary heart disease” to obtain CHD targets. And the targets obtained in the GeneCards database were screened according to the median relevance score. The intersection of the targets of GGD compound and CHD are the potential therapeutic targets of GGD for CHD.

### 2.3. Protein-Protein Interactions

The STRING 11.5 database (https://string-db.org/) was used to analyze the PPI (Protein-Protein Interactions) network of potential therapeutic targets of GGD compounds and visualized by Cytoscape 3.7.2. Among them, the target organism was set to Homo sapiens, and the minimum required interaction score of the network was 0.7. The degree represents the number of nodes in the network that directly interact with the nodes. Betweenness centrality (BC) reflects the importance of the node's transmission in the network. Closeness Centrality (CC) reflects how close a node is to other adjacent nodes. Therefore, in the PPI network, we combine the degree, BC, and CC values to filter the core targets.

The GEO database (https://www.ncbi.nlm.nih.gov/geo/), which stores high-throughput gene expression data in humans, was used to validate the specificity and accuracy of core targets. We used “coronary heart disease” as the keyword and selected “Homo sapiens” as the species to screen suitable datasets for validation. GraphPad Prism software (Inc., La Jolla, CA, USA) was used for the analysis of sample data and visualization of results. *P* values <0.05 for targets were considered statistically significant.

MCODE can find out key sub-networks based on the relationship between nodes and edges in the network to further analyze the biological role of potential targets. Metascape (https://metascape.org) [16] was used for MCODE analysis of PPI networks.

### 2.4. Functional Classification of Potential Targets of GGD Compounds

The PANTHER (Protein ANalysis THrough Evolutionary Relationships) Classification System (https://www.pantherdb.org) [17] can classify proteins and genes according to family, molecular function, biological process, and pathway, to facilitate high-throughput analysis. The potential therapeutic targets of GGD were input into the PANTHER Classification System for functional annotation and classification, and its target organization selected Homo sapiens.

### 2.5. Enrichment Analysis

To further analyze the therapeutic mechanism of GGD compounds for CHD, we performed enrichment analysis including GO biological process analysis and KEGG pathway analysis by the DAVID 6.8 database (https://david.ncifcrf.gov/summary.jsp) [18]. Enrichment analysis results with *P*-value < 0.05 were considered significant and used for further analysis.

### 2.6. Molecular Docking

We used molecular docking to verify the binding of the core targets to GGD compounds. Affinity less than 0 indicates that the ligand and receptor can bind spontaneously. The lower affinity, the better the binding ability. The molecular structures of GGD compounds in mol2 format were from the TCMSP database (https://tcmsp-e.com/) [19]. The crystal structures of the core targets in pdb format were from the RCSB Protein Data Bank (PDB database, (https://www.rcsb.org/) [20], whose screening criteria were determined according to the resolution of protein and whether they have ligands. PyMol 2.5.2 was used to calculate root mean square deviation (RMSD). The docking process was considered reliable when RMSD of the molecular docking results of the ligands was less than 2 [21, 22]. Therefore, we selected EGFR (PDB ID:4RJ3), MAPK3 (PDB ID:6GES), RELA (PDB ID:6HL6), CCND1 (PDB ID:2W96), ESR1 (PDB ID:5ACC), PTGS2 (PDB ID:5IKR), NR3C1 (PDB ID:1NHZ), CYP3A4 (PDB ID:6MA8), and PTPN11 (PDB ID:7JVN) for further processing. AutoDockTools 1.5.6 and AutoDock Vina 1.1.2 were used to simulate the binding of the core targets to the compounds.

## 3. Results

### 3.1. Main Active Compounds of GGD and Their Pharmacological Properties

**Figure 1:**
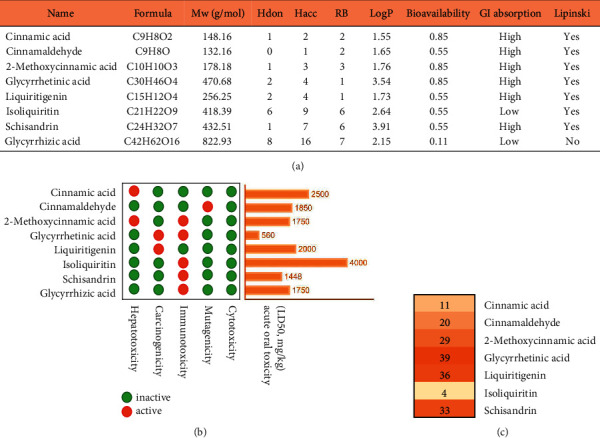
Main active ingredients of Guizhi Gancao decoction (GGD) and their evaluation of pharmacological and toxicological parameters. (a) Physicochemical properties of active compounds in GGD. (b) The toxicological parameters of the active compounds in GGD. (c) Distribution of CHD-related targets in GGD compounds.

Based on UPLC-MS/MS studies, we obtained 8 active compounds of GGD [9]. The RO5 principle is the key to evaluating compounds. According to the prediction of SwissADME, glycyrrhizic acid is not in conformity with the RO5 principle and is not considered for further evaluation as an active ingredient in the treatment of CHD. Cinnamic acid, cinnamaldehyde, 2-Methoxycinnamic acid, glycyrrhetinic acid, liquiritigenin, isoliquiritin, and schisandrin are all in conformity with the RO5 principle (Figure 1(a)).

Drug toxicology is the key to preclinical research. Compounds toxicity (hepatotoxicity, carcinogenicity, immunotoxicity, mutagenicity, cytotoxicity, and acute oral toxicity (LD50)) was predicted by The ProTox-II webserver. None of the compounds exhibited cytotoxicity. Cinnamic acid and 2-Methoxycinnamic acid may be hepatotoxic, glycyrrhetinic acid and liquiritigenin may be carcinogenic, 2-Methoxycinnamic acid, glycyrrhetinic acid, isoliquiritin, schisandrin, glycyrrhizic acid may be immunotoxic, and cinnamaldehyde may be mutagenic (Figure 1(b)). In addition, glycyrrhetinic acid showed the lowest LD50 value (560 mg/kg).

### 3.2. Data Collection of the Targets

Canonical SMILES of GGD effective compounds retrieved from the PubChem database were imported into SwissTargetPrediction, and the resulting targets were screened according to the median of probability values and pooled, yielding 196 targets. At the same time, with the keyword “coronary heart disease,” we obtained 1225 and 3847 targets in the HERB and GeneCards databases, respectively. After all CHD targets were pooled, we obtained 4161 CHD-related targets. We intersected the targets of GGD compounds with CHD-related targets and obtained 131 targets, which may be potential therapeutic targets of GGD for CHD. Supplementary Table S1 provides detailed information on 131 targets.  Figure 1(c) shows the number of targets of each compound, glycyrrhetinic acid has the most potential targets related to CHD (39 targets), followed by liquiritigenin (36 targets) and schisandrin (33 targets), which suggested that these compounds may be key compounds for GGD treatment of CHD.

### 3.3. Protein-Protein Interactions

**Figure 2:**
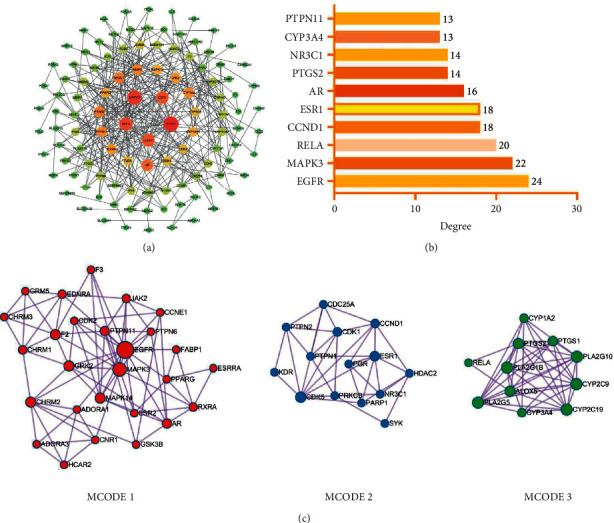
PPI Network: (a) The PPI network was constructed for 131 potential therapeutic targets of GGD to CHD. The size and color of nodes are related to the degree. (b) The top ten targets in the PPI network by degree, BC and CC values value. (c) The first three clusters of MCODE analysis.

To explore GGD compound interactions and their core targets, we constructed PPI networks through the STRING 11.5 database and visualized them through Cytoscape 3.7.2. Therefore, 131 CHD-related GGD compound targets were constructed as a PPI network with 111 nodes and 330 edges (Figure 2(a)). EGFR, MAPK3, RELA, CCND1, ESR1, AR, PTGS2, NR3C1, CYP3A4, and PTPN11 may be the core targets, which were filtered by degree, BC, and CC values (Figure 2(b) and Table 1). Among them, EGFR has the highest degree (degree = 24), followed by MAPK3 (degree = 22) and RELA (degree = 20).

**Figure 3:**
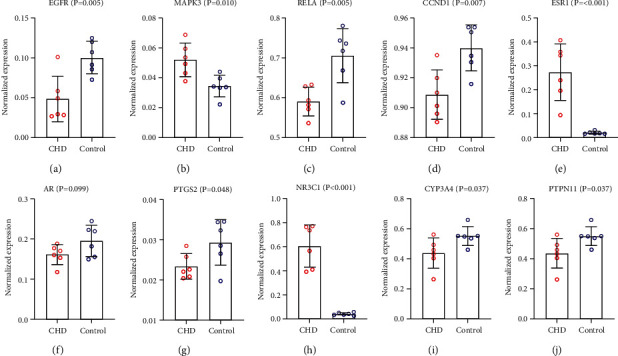
Differential expression analysis of core targets in the chip dataset GSE62867.

The chip dataset GSE62867 in the GEO database contains 6 coronary atherosclerotic plaque samples and 6 whole blood samples, which were used to validate the differential analysis of core targets. GraphPad Prism was used for the analysis of sample data and visualization of results (Figure 3). Validated by the GEO dataset, EGFR, MAPK3, RELA, CCND1, ESR1, PTGS2, NR3C1, CYP3A4, and PTPN11 have significant differential expression and were considered to be the core targets of GGD for the treatment of CHD.

Then, we used Metascape to perform MCODE analysis on the PPI network and obtained 6 clusters, Supplementary Table S2 showed the specific information of each cluster, and we visualized the first three clusters by Cytoscape 3.7.2 (Figure 2(c)). The pathway and process enrichment analysis of each MCODE cluster was shown in Table 2. The PI3K-Akt signaling pathway is a potential therapeutic mechanism of MCODE 1 for CHD, and EGFR is a key target of MCODE 1. The mechanism of the therapeutic of MCODE 2 on CHD is by regulating thyroid hormone and cell cycle, and its key target is CDK5. Arachidonic acid and linoleic acid metabolism may be the therapeutic mechanism of MCODE 3 for CHD and the key target of MCODE 3 is CYP2C9.

### 3.4. Functional Classification of Potential Targets of GGD Compounds

**Figure 4:**
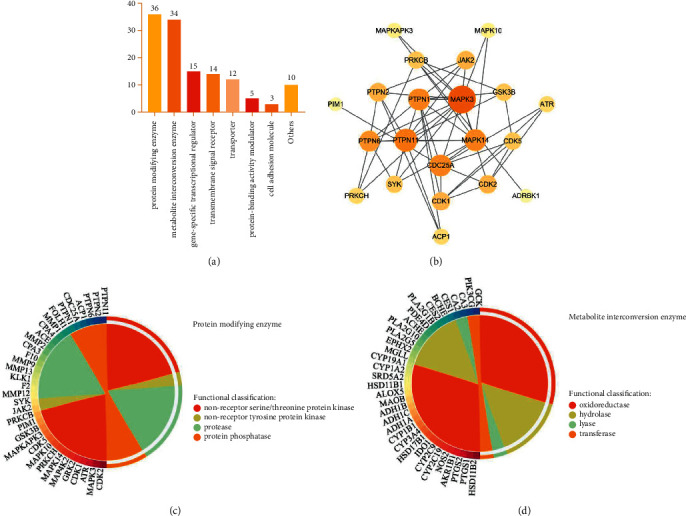
Functional classification of potential therapeutic targets for GGD. (a) Panther classification. (b) PPI network is composed of protein kinases and protein phosphatases. The size and color of a node are related to the degree. (c, d) Classification of proteins involving protein modifying enzyme (PC00260) and metabolite interconversion enzyme (PC00262).

Functional classification of potential targets of GGD compounds was performed by the PANTHER Classification System. According to protein class, potential targets of GGD compounds were divided into 8 classes (Figure 4(a)), of which protein modifying enzyme (PC00260, 36 targets) is the largest category, followed by metabolite interconversion enzyme (PC00262, 34 targets) and gene-specific transcriptional regulator (PC00264, 15 targets).

Protein kinases are involved in the process of catalyzing protein phosphorylation and play an important role in regulating protein activity and signal transduction. Protein phosphatases catalyze the process of protein dephosphorylation. The two work together to regulate the phosphorylation/dephosphorylation of proteins, which is crucial in cellular signal transduction. Among protein-modifying enzymes, 14 targets were non-receptor serine/threonine protein kinase, 2 targets were non-receptor tyrosine-protein kinase, and 6 targets were protein phosphatase. They form a complex PPI network, which has 21 nodes and 52 edges (Figure 4(b)). Among them, MAPK3 and PTPN11 play key roles. For other protein modification enzymes, MMP12, F2, KLK1, MMP13, MMP9, F10, CPA3, MMP2, ACE, CPA4, MMP1, and FOLH1 belong to protease (Figure 4(c)). For the metabolite interconversion enzyme, 20 targets were oxidoreductase, 10 targets were hydrolase, 2 targets were lyase, and 2 targets were transferase (Figure 4(d)). It can be seen that the potential therapeutic targets of GGD compounds for CHD involve multiple key biological processes in the human body.

### 3.5. Enrichment Analysis

A total of 131 potential therapeutic targets of GGD were entered into DAVID 6.8 database for GO analysis and KEGG pathway analysis, in which target identifier was set as gene symbol and target species was set as Homo sapiens.

**Figure 5:**
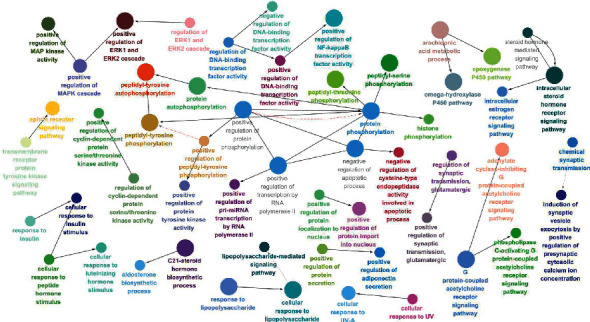
Enrichment analysis of GO biological process of potential therapeutic targets for GGD.

In the GO enrichment, 284 of the 386 terms in the biological process (BP) analysis had a *P*-value <0.05. The ClueGo feature of Cytoscape 3.7.2 was used to visualize the BP analysis (Figure 5). Potential targets of GGD for the therapeutic mechanism of CHD involve various biological processes, such as protein phosphorylation, inflammatory response, signal transduction, intracellular steroid hormone receptor signaling pathway, and negative regulation of apoptosis.

**Figure 6:**
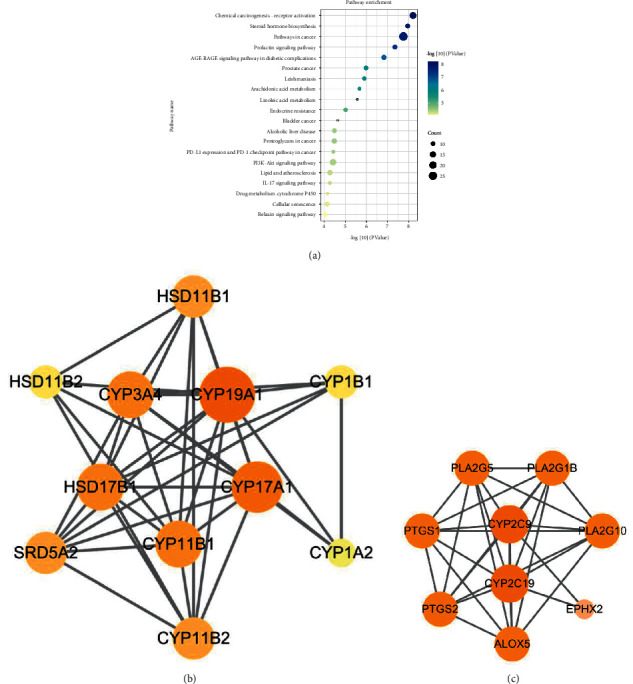
Enrichment analysis of KEGG of potential therapeutic targets for GGD. (a) The top 20 pathways of KEGG analysis. (b, c) PPI network of steroid hormone biosynthesis metabolism pathway, arachidonic acid pathway. The size and color of a node are related to the degree.

A total of 119 of the 134 pathways analyzed by the KEGG pathway had a *P*-value of <0.05. We show the top 20 pathways (Figure 6(a)). And steroid hormone biosynthesis metabolism, arachidonic acid metabolism, linoleic acid metabolism, PI3K-Akt signaling pathway, lipid and atherosclerosis, IL-17 signaling pathway, relaxin signaling pathway may be potential therapeutic mechanisms of GGD for CHD. Figure 6(b) is a PPI network for steroid hormone biosynthesis metabolism pathway based on 11 GGD targets, with 11 nodes and 39 edges. Among them, CYP19A1 is the core target. In addition, the 9 GGD targets involved in the arachidonic acid metabolism pathway constitute a PPI network with 9 targets and 30 edges (Figure 6(c)). Among them, CYP2C9 and CYP2C19 are the core targets.

### 3.6. Molecular Docking

AutoDockTools 1.5.6 and AutoDock Vina 1.1.2 were used to verify the binding of the core targets to GGD compounds and the lowest energy docking model was selected. Studies have shown that the affinity is lower than−4.25 kcal/mol has a certain binding force, the affinity is lower than−5.0 kcal/mol with a good binding force, and the affinity is lower than−7.0 kcal/mol, which has a strong binding force [23, 24].

**Figure 7:**
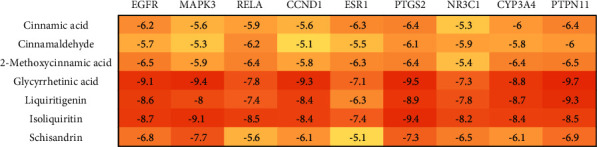
Molecular docking results of GGD active compounds and core targets.

**Figure 8:**
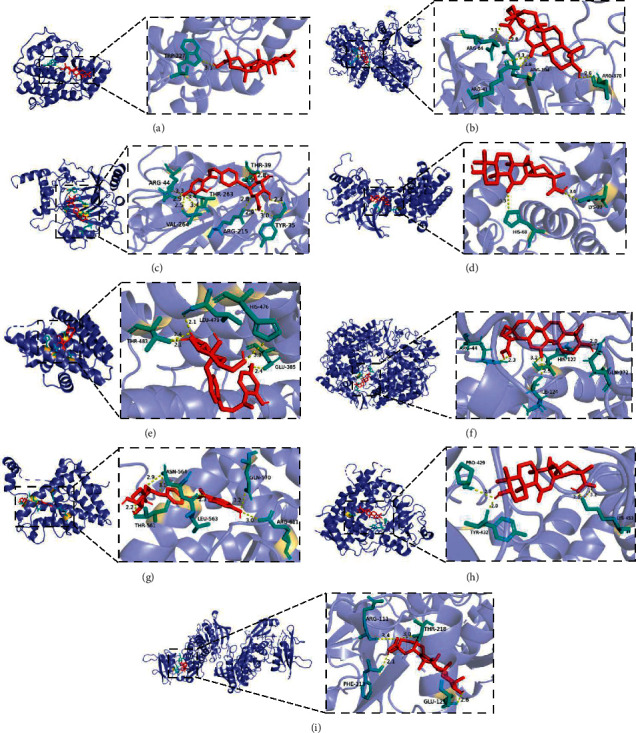
Targets with the highest affinities in molecular docking. (a) Glycyrrhetinic acid and EGFR, affinity = −9.1 kcal/mol. (b) Glycyrrhetinic acid and MAPK3, affinity = −9.4 kcal/mol. (c) Isoliquiritin and RELA, affinity = −8.5 kcal/mol. (d) Glycyrrhetinic acid and CCND1, affinity = −9.3 kcal/mol. (e) Isoliquiritin and ESR1, affinity = −7.4 kcal/mol. (f) Glycyrrhetinic acid and PTGS2, affinity = −9.5 kcal/mol. (g) Isoliquiritin and NR3C1, affinity = −8.2 kcal/mol. (h) Glycyrrhetinic acid and CYP3A4, affinity = −8.8 kcal/mol. (i) Glycyrrhetinic acid and PTPN11, affinity = −9.7 kcal/mol.

**Figure 9:**
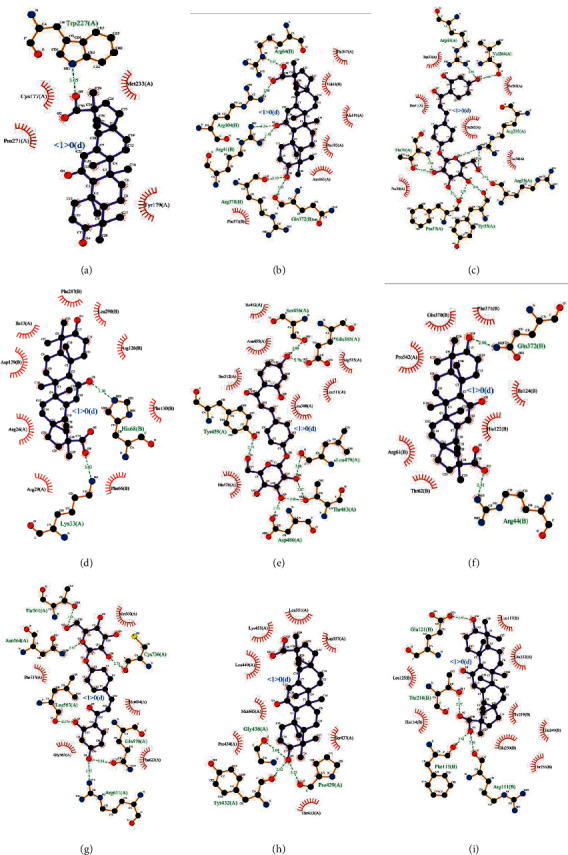
Interactions between compounds and residues in protein binding sites. (a) Glycyrrhetinic acid and EGFR. (b) Glycyrrhetinic acid and MAPK3. (c) Isoliquiritin and RELA. (d) Glycyrrhetinic acid and CCND1. (e) Isoliquiritin and ESR1. (f) Glycyrrhetinic acid and PTGS2. (g) Isoliquiritin and NR3C1. (h) Glycyrrhetinic acid and CYP3A4. (i) Glycyrrhetinic acid and PTPN11.

The results showed that all core targets bind well to GGD compounds, and almost all key compounds show a strong binding ability to core targets (Figure 7). We showed the targets with the highest affinities (Figure 8) and presented that all interacting residues interact with the protein-ligand complex (Figure 9), which further suggested that GGD compounds may play an essential role in the treatment of CHD.

## 4. Discussion

CHD is the leading cause of death from CVD and has become an important public health problem worldwide. GGD has been widely used for a long time in the treatment of various CVD including CHD and heart failure and has a significant effect on protecting the heart [6, 7]. Our study shows that glycyrrhetinic acid, liquiritigenin, and schisandrin are key compounds in the GGD treatment of CHD. The therapeutic mechanism of GGD for CHD may be related to steroid hormone biosynthesis metabolism, arachidonic acid metabolism, linoleic acid metabolism, PI3K-Akt signaling pathway, lipid and atherosclerosis, IL-17 signaling pathway, and relaxin signaling pathway.

Atherosclerosis is the root cause and complication of most CVDs such as CHD, and myocardial infarction. CHD is characterized by atherosclerosis of the coronary arteries. Under normal conditions, arterial endothelial cells resist leukocyte adhesion, but when abnormally stimulated (dyslipidemia, pro-inflammatory mediators), endothelial permeability and extracellular matrix components are altered and induce leukocyte adhesion, resulting in the accumulation of intracellular cholesterol [25]. The accumulation of lipid-laden macrophages under the arterial endothelium is a hallmark of atherosclerosis [26]. Lipid metabolism and atherosclerosis have always been the focus of CHD research, and it has become a consensus to manage lipid levels in patients with CHD and use statins and other lipid-lowering drugs [27–29]. However, with further research, our understanding of CHD has been updated. Atherosclerosis is not only the result of disturbances in lipid metabolism but is defined as a chronic inflammatory disease. Lipid-rich cells under the arterial endothelium promote inflammatory responses in the arterial wall, leading to pathological processes such as plaque formation, rupture, and hemorrhage [26, 30].

Polyunsaturated fatty acids (PUFAs) are involved in the regulation of inflammation, immunity, and vascular function, and are key factors in CVD [31]. At present, replacing saturated fat with PUFAs is currently one of the important recommendations in global dietary guidelines to reduce CHD risk [32, 33]. Among them, arachidonic acid (AA) and linoleic acid (LA) is the focus of research. The AA metabolism pathway is closely related to pathological processes including oxidative stress, inflammation, vascular homeostasis, and apoptosis [34, 35]. AA is a precursor to many pro-inflammatory/pro-aggregating substances such as thromboxane, leukotriene prostaglandins, and other oxidized derivatives and is a central component of the inflammatory response. Among them, prostaglandin E2 (PGE2), leukotriene B4 (LTB4) and thromboxane A2 (TXA2) are the most critical inflammatory and aggregation mediators in AA-derived eicosanoids [36]. Specifically, PGE2 can promote the production of itself and the inflammatory factor IL-6, and increase vascular permeability. LTB4 can promote the production of inflammatory factors IL-1*β*, IL-6, and TNF-*α*, increase vascular permeability and promote the chemotaxis and adhesion of leukocytes. TXA2 can promote platelet activation and aggregation and is involved in thrombosis and atherosclerosis [37, 38]. COX is a key enzyme in the metabolism and derivatization of AA. COX-1 is involved in the production of TXA2 and is generally constitutively expressed. Experiments have shown that significant downregulation of the AA/COX-1/TXB2 (Stable metabolite of TXA2) pathway exhibits antiplatelet effects [39]. Aspirin (COX-1 inhibitor) has been widely used in CHD patients. The expression of COX-2 is induced by various factors such as cytokines, inflammatory factors, ischemia, and hypoxia. COX-2 is involved in immune regulation and angiogenesis and inhibits apoptosis. Studies support that COX-2 regulates atherosclerotic plaque stability and endothelial function in CHD patients [40, 41]. In addition, eicosapentaenoic acid (EPA), as a key anti-inflammatory/anti-aggregation long-chain PUFA, its ratio to AA (EPA/AA) was used as risk stratification for CHD patients [38, 42].

AA is the major metabolite of LA. Among vegetable oils, only LA reduces serum cholesterol levels [43]. Therefore, the positive effect of LA on CHD is considered to be achieved by regulating blood lipid levels. In dietary recommendations for replacing saturated fat with PUFAs to reduce CHD risk, it is essentially a recommendation to increase LA intake [33]. Although AA, which is closely involved in the inflammatory response, is the major metabolite of LA, evidence suggests that increasing LA intake does not increase the concentration of excessive inflammatory markers and may even attenuate the inflammatory response [37]. Furthermore, LA intake was shown to be inversely related to CHD risk [44].

Oxidized derivatives of LA affect adrenal steroid metabolism and mediate obesity and oxidative stress [45]. Steroid hormones are synthesized from cholesterol and are divided into sex hormones and corticosteroid hormones, which play an important role in maintaining life, immune regulation, and body development. Various steroid hormones can activate HSF-1 and upregulate HSP72 in adult male myocardium to protect the heart [46]. Serum androgen levels are inversely associated with coronary heart disease risk in adult males [47]. Oxidized derivatives of LA stimulate aldosterone production and inhibit aldosterone production at higher doses [45]. And aldosterone is an important endogenous mineralocorticoid in the human body. It upregulates the expression of low-density lipoprotein receptor 1 (LOX-1) [48], mediates vascular uptake of oxidized LDL, and participates in endothelial dysfunction, foam cell formation, and plaque instability [49], which plays a key role in atherosclerosis. In addition, aldosterone reduced the activity of endothelial glucose-6-phosphate dehydro-genase (G6PD), attenuated antioxidant capacity and availability of NO, which affects the metabolism of AA, and activated NF-kB in the heart, leading to an increase in inflammatory cytokines [50–52]. Studies have supported the use of aldosterone inhibitors to show beneficial effects on atherosclerosis and cardiac function [48, 53]. Aldosterone was significantly associated with CVD and mortality [54] and significantly modulates platelet responsiveness to arachidonate, reducing the pro-aggregatory potential of arachidonate [55].

The PI3K-Akt signaling pathway is closely related to the pathological process of atherosclerosis and CHD. Oxidized LDL activates PI3K in macrophages, which is widely expressed in the cardiovascular system, resulting in the production of the second messenger PIP3 which in turn activates the downstream factor Akt of PI3K through phosphorylation [56, 57]. Activation of Akt can inhibit apoptosis and regulate cell proliferation, differentiation, and autophagy through downstream target proteins such as mTOR, NF-*κ*B, and eNOS. Activation of the PI3K/Akt signaling pathway has been confirmed to reduce lipid deposition, inhibit apoptosis, and resist atherosclerosis [58, 59]. Moreover, the PI3K/Akt signaling pathway could regulate the expression of inflammatory factors in cardiomyocytes and was associated with the degree of CHD [60].

The PI3K/Akt signaling pathway can regulate the expression of IL-17 [61], which induces the expression of cytokines and chemokines and plays a key role in acute and chronic inflammation [62]. Experiments have shown that IL-17 plays a significant role in promoting inflammation and promoting atherosclerosis in ApoE−/− mice [63, 64]. Furthermore, differential expression of IL-17 was found in human atherosclerotic plaques [65]. Elevated plasma IL-17 levels have been found in patients with unstable angina, acute myocardial infarction, and acute coronary syndrome [66]. In addition, IL-17 induces cell death of vascular endothelial cells, which in turn alters plaque stability [67], and it is thought to synergize with TNF-*α* to promote the development of CHD [68].

Our research suggests that glycyrrhetinic acid, liquiritigenin, and schisandrin are key compounds in the GGD treatment of CHD. Molecular docking results show that the affinity values of these components are not only less than 0 but also have high binding capacity. This shows that they can spontaneously bind to the corresponding targets, and the binding ability is good.

Flavonoids have been shown to have clear potential cardiovascular benefits, and increasing flavonoid intake can effectively reduce CVD risk [69]. Liquiritigenin belongs to the class of flavonoids with anti-inflammatory, antioxidant, and estrogenic properties. Experiments show that liquiritigenin can reduce ROS generation and lipid accumulation, down-regulate inflammatory response, and improve cardiac function [70–72]. In addition, liquiritigenin was shown to attenuate myocardial fibrosis and inhibit myocardial apoptosis through Akt/ERK signaling pathway [73].

Glycyrrhetinic acid is formed by the hydrolysis of glycyrrhizic acid to remove the sugar acid chain and is widely distributed in licorice roots. Glycyrrhetinic acid has an obvious protective effect on cardiovascular, and experiments show that it can regulate the permeability of rat heart mitochondria and mediate cardiac oxidative stress and apoptosis, and improve MIRI [74, 75]. Moreover, glycyrrhetinic acid can affect myocardial muscle strength, inhibit oxidative stress, reduce the inflammatory response, and protect the heart through PI3K/Akt pathway [76, 77].

Schisandrin has multiple pharmacological activities and has been shown to have antioxidant, anti-inflammatory, and anti-ER stress effects [78]. Evidence supports that Schisandrin protects the heart by reducing oxidative stress, inhibiting apoptosis, and improving MIRI [78, 79].

In this study, the active compounds of GGD were obtained based on UPLC-MS/MS, which avoided the use of public databases to obtain a large number of non-specific components. In addition, we identified the mechanism and active compounds of GGD in the treatment of CHD, which provided preliminary evidence for the therapeutic potential of GGD for CHD, and provided candidate compounds for anti-CHD. This is beneficial to the clinical treatment of CHD and the development of new drugs.

However, this study has some limitations. First, due to the limitations of network pharmacology, we could not consider the effect of GGD dose on CHD. Second, because the current network is static, it contradicts the pathophysiological process of the dynamic development of the human body. Finally, although experimental studies have shown that GGD has a good therapeutic effect on CHD, the research on the active ingredients and specific mechanism of GGD remain unclear, and whether the active compounds of GGD have synergistic effects needs further exploration in experimental and clinical studies.

## 5. Conclusions

According to the network pharmacology method, we determined the therapeutic mechanism and material basis of GGD for CHD. And molecular docking demonstrated the effectiveness of these compounds with their targets, providing preliminary evidence for their potential in the treatment of CHD. Moreover, this further provides candidate compounds for the treatment of CHD, which is beneficial to the development of new drugs and clinical treatment. However, its clinical application still needs further research and confirmation.

## Figures and Tables

**Table 1:** The core targets of GGD in the treatment of CHD, which are screened by degree, Betweenness centrality (BC), and Closeness centrality (CC) values.

Name	Degree	Betweenness centrality	Closeness centrality
EGFR	24	0.16658958	0.46753247
MAPK3	22	0.13365082	0.46153846
RELA	20	0.10815601	0.43548387
ESR1	18	0.11053722	0.44262295
CCND1	18	0.07766806	0.41221374
AR	16	0.08484615	0.41860465
PTGS2	14	0.10940925	0.42352941
NR3C1	14	0.04845664	0.421875
PTPN11	13	0.04900512	0.40298507
CYP3A4	13	0.09516744	0.35179153

**Table 2:** The pathway and process enrichment analysis of the MCODE cluster.

MCODE	GO	Description	Log10 (P)
MCODE 1	hsa05200	Pathways in cancer	−14
MCODE 1	hsa04151	PI3K-Akt signaling pathway	−10.8
MCODE 1	hsa04080	Neuroactive ligand-receptor interaction	−10.7
MCODE 2	hsa05207	Chemical carcinogenesis - receptor activation	−7.5
MCODE 2	hsa04919	Thyroid hormone signaling pathway	−6.6
MCODE 2	hsa04110	Cell cycle	−6.6
MCODE 3	hsa00590	Arachidonic acid metabolism	−19.5
MCODE 3	hsa00591	Linoleic acid metabolism	−18.9
MCODE 3	hsa05204	Chemical carcinogenesis - DNA adducts	−10.6

## Data Availability

The data used to support the findings of this study are included within the article and supplementary materials.

## Supplementary Materials

Supplementary Materials Supplementary Table S1. Core targets of GGD and their corresponding active compounds. Supplementary Table S2. MCODE analysis of the PPI network.
