# Supplementary material for: To Explore the Key Active Compounds and Therapeutic Mechanism of Guizhi Gancao Decoction in Coronary Heart Disease by Network Pharmacology and Molecular Docking
Source: Evid Based Complement Alternat Med. 2022 Nov 23;2022:2566407. doi: 10.1155/2022/2566407 (PMC9711953; doi:10.1155/2022/2566407)
Supplement: Supplementary Materials — Supplementary Table S1. Core targets of GGD and their corresponding active compounds. Supplementary Table S2. MCODE analysis of the PPI network. [file 2566407.f1.zip › Supplementary Table S1.pdf]

Supplementary Table S1: Core targets of GGD and their corresponding active compounds.

Compound	Target
Cinnamaldehyde	TRPA1, HCAR2, CA2, CHRNA7, GSK3B, TLR4, NOS2, F3, ADH1B, ADH1C, ADH1A, CHRM2, CHRM1, CHRM3, RELA, MGLL, HDAC2, PARP1, CYP11B1, CYP11B2
Cinnamic acid	HCAR2, CA2, AKR1B1, SLC16A1, CA3, TRPA1, ALOX5, MMP9, MMP1, MMP2, PTPN1
2-Methoxycinnamic acid	CA2, AKR1B1, TLR4, ESR2, SLC16A1, PTGS2, MAOB, HSD17B1, CYP2C9, CYP3A4, CYP2C19, HCAR2, CA3, MMP9, MMP1, MMP2, MMP13,CYP1A2, EGLN3, FOLH1, CPA3, SLC22A12, KDM3A, ACE, ITGAL, ICAM1, ITGB2, GRK2, PLEC
Glycyrrhetinic acid	PTPN1, CES2, NOS2, CYP19A1, SHBG, BCHE, ALOX5, POLB, ADORA3, ESR2, PLA2G1B, CES1, PPARG, ESR1, PRKCH, HSD11B2, PTPN11, HSD11B1, AR, PTPN2, NPC1L1, SERPINA6, CYP17A1, PGR, NR3C1, CDC25A, RORA, MAPK3, PTPN6, NR1H3, IDO1, ACP1, SRD5A2, PDE4D, FABP1, NR3C2, NR1I2, SCD, NR1I2
Liquiritigenin	CYP19A1, HSD17B1, ESR1, ESR2, SHBG, MAOB, ADORA3, PLA2G1B, CES1, PPARG, CES2, MMP13, POLB, BCHE, CHRNA7, CA2, CA3, ABCC1, CYP1B1, PTGS1, ADORA1, ABCG2, MMP12, SLC5A2, PLA2G5, PLA2G10, GRM5, SERPINE1, RXRA, EDNRA, KLK1, ESRRA, ESRRB, DYRK1B, ACHE, DYRK1A
Isoliquiritin	AKR1B1, PTPN1, SLC29A1, EPHX2
Schisandrin	ALOX5, PTGS2, GSK3B, CYP2C9, CYP3A4, CYP2C19, ATR, GCK, NTRK1, MDM2, JAK2, EGFR, SYK, CDK1, MAPK14, MAPK10, BRD4, CDK5, KDR, CNR1, CCND1, CDK2, CCNE1, CSNK2A1, ABCC9, F2, F10, TYRO3, P2RX3, PIM1, P2RX7, PRKCB, PIK3CG
