# Supplementary material for: To Explore the Key Active Compounds and Therapeutic Mechanism of Guizhi Gancao Decoction in Coronary Heart Disease by Network Pharmacology and Molecular Docking
Source: Evid Based Complement Alternat Med. 2022 Nov 23;2022:2566407. doi: 10.1155/2022/2566407 (PMC9711953; doi:10.1155/2022/2566407)
Supplement: Supplementary Materials — Supplementary Table S1. Core targets of GGD and their corresponding active compounds. Supplementary Table S2. MCODE analysis of the PPI network. [file 2566407.f1.zip › Supplementary Table S2.pdf]

Supplementary Table S2: MCODE analysis of the PPI network

Cluster	Score	Type	Gene	Symbol
1	3	Clustered	2152	F3
1	3	Clustered	5468	PPARG
1	3	Clustered	134	ADORA1
1	3	Clustered	3717	JAK2
1	3	Clustered	1909	EDNRA
1	3	Clustered	898	CCNE1
1	3	Clustered	140	ADORA3
1	3	Clustered	1131	CHRM3
1	3	Clustered	367	AR
1	3	Clustered	2147	F2
1	3	Seed	5781	PTPN11
1	3	Clustered	5777	PTPN6
1	3	Clustered	1129	CHRM2
1	3	Clustered	1017	CDK2
1	3	Clustered	1128	CHRM1
1	3	Clustered	156	GRK2
1	3	Clustered	2100	ESR2
1	3	Clustered	338442	HCAR2
1	3	Clustered	1432	MAPK14
1	3	Clustered	5595	MAPK3
1	3	Clustered	1268	CNR1
1	3	Clustered	1956	EGFR
1	3	Clustered	2168	FABP1
1	3	Clustered	6256	RXRA
1	3	Clustered	2932	GSK3B
1	3	Clustered	2101	ESRRA
1	3	Clustered	2915	GRM5
2	2.357143	Clustered	983	CDK1
2	2.357143	Clustered	2908	NR3C1
2	2.357143	Clustered	5579	PRKCB
2	2.357143	Clustered	595	CCND1
2	2.357143	Clustered	5770	PTPN1
2	2.357143	Clustered	5241	PGR
2	2.357143	Clustered	142	PARP1
2	2.357143	Clustered	5771	PTPN2
2	2.357143	Seed	993	CDC25A
2	2.357143	Clustered	2099	ESR1
2	2.357143	Clustered	3066	HDAC2
2	2.357143	Clustered	1020	CDK5
2	2.357143	Clustered	3791	KDR
2	2.357143	Clustered	6850	SYK
3	3.727273	Clustered	5319	PLA2G1B
3	3.727273	Clustered	240	ALOX5
3	3.727273	Seed	5742	PTGS1
3	3.727273	Clustered	1544	CYP1A2
3	3.727273	Clustered	1559	CYP2C9
3	3.727273	Clustered	5322	PLA2G5
3	3.727273	Clustered	1576	CYP3A4
3	3.727273	Clustered	5970	RELA
3	3.727273	Clustered	5743	PTGS2
3	3.727273	Clustered	1557	CYP2C19
3	3.727273	Clustered	8399	PLA2G10
4	1.4	Seed	4313	MMP2
4	1.4	Clustered	3689	ITGB2
4	1.4	Clustered	3290	HSD11B1
4	1.4	Clustered	4318	MMP9
4	1.4	Clustered	4312	MMP1
5	1.5	Clustered	1585	CYP11B2
5	1.5	Seed	6716	SRD5A2
5	1.5	Clustered	1588	CYP19A1
5	1.5	Clustered	1584	CYP11B1
6	1.5	Clustered	126	ADH1C
6	1.5	Clustered	125	ADH1B
6	1.5	Seed	124	ADH1A
6	1.5	Clustered	4129	MAOB
